# Socioeconomic factors and out-of-pocket healthcare spending among older adults with hypertension and type 2 diabetes in the US: findings from the 2018–2022 MEPS data

**DOI:** 10.1186/s12913-026-15188-5

**Published:** 2026-07-25

**Authors:** Tonghui Xu, Alfonzo E. Munoz, Thai A. Tran, Omolola E. Adepoju

**Affiliations:** 1 Humana Integrated Health Systems Sciences Institute, Houston, TX USA; 2 Tilman J Fertitta Family College of Medicine, University of Houston, Houston, TX USA

**Keywords:** Out-of-pocket medical expenditure, Diabetes and hypertension, MEPS data, Social determinant of health, National data analysis

## Abstract

**Background:**

Type 2 diabetes and hypertension often co-occur significantly, adding to patients’ healthcare economic expenditure, especially among older adults. This study examined socioeconomic factors associated with annual combined total out-of-pocket (OOP) healthcare expenditures for type 2 diabetes and hypertension among adults aged 50 years and older with co-occurring hypertension and diabetes in the United States.

**Method:**

This secondary data analysis used pooled, nationally representative data from the 2018–2022 Medical Expenditure Panel Survey (MEPS) and focused on U.S. adults aged 50 years and older with type 2 diabetes and hypertension. The outcome variable was the combined annual OOP cost for both type 2 diabetes and hypertension among patients with both conditions. Covariates include age, gender, race/ethnicity, insurance coverage (e.g., Medicare, Medicaid/TRICARE, private, or more than one insurance type), annual family income, region, employment status, education, marital status, perceived general health, and perceived mental health. Because the outcome variable was right-skewed, we performed a logarithmic transformation to normalize the OOP cost variable. We then applied a survey weighted linear regression model (with the log-transformed outcome) to examine the relationship between sociodemographic variables and the combined OOP medical expenditures. The model accounted for the complex sampling design of the MEPS data, including stratification, clustering, weighting structure, and inflation adjustments (reported in December 2022 U.S. dollars using the Consumer Price Index [CPI] Inflation Calculator). All analyses were conducted using SAS 9.4.

**Results:**

We included 7,341 individuals with both conditions. The average weighted annual OOP costs among adults with both conditions were $474.95, $413.16 for diabetes specific expenditures, and $84.37 for hypertension specific expenditures. Adults residing in the Northeast who were aged 65 years and older, non-Hispanic White, married, had higher income, had a high school education or higher, reported poor perceived health status, and were either uninsured or had private insurance had significantly higher odds of higher OOP costs.

**Conclusions:**

These findings underscore the complex interplay of health status, socioeconomic factors, and insurance coverage in driving differences in annual OOP healthcare expenditures among adults aged 50 and older with both hypertension and type 2 diabetes in the United States.

## Introduction

Type 2 diabetes and hypertension remain the two most commonly co-occurring chronic diseases, and their incidence is dramatically increasing worldwide [1]. About 75% of individuals with a diabetes diagnosis also have hypertension, accounting for nearly 23 million adults in the United States [2]. Along with the negative health effects associated with both conditions, a financial burden is also associated with their continuous management, medications, and the need to monitor them constantly [3, 4]. For example, the total healthcare costs of hypertension were estimated to range from approximately $15.0 billion to $54.0 billion in 2001 [5]. A national estimate showed that diabetes remained one of the costliest chronic conditions in the United States, with estimated medical expenditures totaling $412.9 billion in 2022 [6]. A previous study estimated that the national burden of comorbid diabetes and hypertension could exceed $350 billion [7]. This trend is especially alarming in middle-aged and older adults because the prevalence of these conditions increases with age [8, 9].

For individuals with diabetes or hypertension, high medical expenditures are common, especially when these conditions co-occur. Several studies have indicated that individuals with multiple chronic diseases face significant medical expenses, particularly out-of-pocket (OOP) costs [10]. A 2019 study noted that the average economic cost per individual with diagnosed diabetes was $13,240, compared to $4,250 among individuals without diabetes [3]. Earlier studies have also reported higher total healthcare expenditures [11] and OOP medical expenses [7] among patients with co-occurring diabetes and hypertension.

Drawing on the Andersen Behavioral Model of Health Services Use [12], we describe how socioeconomic factors, including income, education, race/ethnicity, and insurance status influence patients’ OOP healthcare burden, through differential access to resources, health coverage, and structural inequities in care. Prior work has identified a correlation between rising OOP healthcare costs for managing chronic conditions, such as diabetes and hypertension [13]. However, a major deficit remains in the literature examining how social determinants, including income, education, or insurance status, compound OOP costs in conjunction with the growing diabetes and hypertension burden [14]. Additionally, much of the existing evidence is based on pre-pandemic data, highlighting the need for more up-to-date research. Analyzing OOP healthcare expenditures in a post-pandemic era, in the context of these social factors, elucidates whether individuals, when given access to care, can afford to adhere to treatment. Therefore, this study examined the socioeconomic factors associated with annual combined OOP healthcare expenditures related to type 2 diabetes and hypertension among adults aged 50 years and older with both conditions in the United States.

## Methods

### Data source

The present study analyzed the 2018–2022 Medical Expenditure Panel Survey (MEPS) datasets. MEPS is a large national survey of families and individuals in the United States, collecting detailed medical, insurance, and demographic information. The survey consists of four components: (a) household, (b) insurance/employer, (c) medical provider, and (d) survey questionnaire [15]. The data files used in this study comprise full-year consolidated panel data, including medical conditions, prescribed medications, hospital inpatient stays, emergency room visits, outpatient visits, office-based medical provider visits, and home health records.

## Study sample

First, we identified type 2 diabetes (International Classification of Diseases [ICD]–10–CM category E11) and hypertension (ICD–10–CM category I10) in the MEPS Medical Conditions File. After excluding individuals younger than 50 years, this study comprised a pooled cross-sectional sample of 7,341 individuals 50 + years old with both type 2 diabetes and hypertension between 2018 and 2022. We restricted our sample to adults aged 50 and older for clinical and policy reasons. The prevalence, complications, and healthcare use related to type 2 diabetes and hypertension increase substantially with age. This age range also captures the transition from private insurance or Medicaid (ages 50–64) to Medicare eligibility at age 65, making it important for understanding healthcare and cost differences across coverage types. Table 2 below reports the detailed characteristics of the sample, categorized by sociodemographic factors.

Next, condition records for type 2 diabetes and hypertension were linked to medical events using the MEPS Condition-Event Link Files. We then calculated event-level OOP expenditures for all medical events linked to E11 and, separately, for all medical events linked to I10, and then aggregated event-level OOP expenditures to the person level. We did not allocate event costs across conditions; if an event was linked to a condition, the full event-level OOP cost was included. For the combined OOP cost estimate, the two condition-specific event lists were pooled, and duplicate person–event records were removed using the DUPERSID–EVNTIDX identifier to avoid double counting events linked to both conditions. As a result, the sum of the diabetes-specific and hypertension-specific OOP costs may be greater than the combined OOP cost estimate. We then linked the combined OOP costs to demographic information and applied survey weights from the Full-Year Consolidated Files, along with the pooled variance PSU (PSU9623) and stratum (STRA9623) variables from the Pooled Linkage File. Condition-specific OOP costs were calculated separately for each condition.

## Measures

The outcome variable, total annual family OOP medical expenditures related to type 2 diabetes and hypertension, was defined as the sum of all annual OOP payments made by individuals or their families for medical events associated with type 2 diabetes and hypertension. The OOP medical expenditure data in our study included the following six event types: (a) home health care, (b) hospital inpatient stays, (c) outpatient visits, (d) emergency room visits, (e) office-based medical provider visits, and (f) prescribed medicines.

Independent variables included (a) demographic characteristics, (b) health status, and (c) insurance. Because some independent variables were collected in multiple rounds within each year, we used values from the first round of each year to ensure that covariates were measured prior to the accumulation of annual medical expenditures. The demographic characteristics included age, categorized into two groups: 50–64 and 65+; education (less than high school, high school, Some college/college degree; biological sex (male or female), race/ethnicity (Asian, Black or African American, non-Hispanic White, Hispanic, and others or two more races), employment status (employed and unemployed), marital status (married, widowed/divorced/ separated, never married), region (Northeast, Midwest, South, and West), family annual income level (poor/near poor: <125% Federal poverty level (FPL), low income: 125%–199% FPL, middle income: 200%–399% FPL, and high income: ≥ 400% FPL). Two health status variables consisted of perceived mental health status (excellent/ very good/good, fair, and poor) and perceived health status (excellent/ very good/good, fair, and poor). Both perceived health statuses were reported by the participants themselves and their family members. Insurance coverage was categorized as Medicare, Medicaid, private insurance, uninsured, and more than one insurance type (e.g., Medicaid and Medicare enrollment).

### Statistical analysis

Descriptive statistics were used to summarize the analytic sample. Since we found that the outcome variable was right-skewed, we first conducted a logarithmic transformation to normalize OOP cost variable. For $0.00 OOP cost, we added $1.00 for each to allow to do logarithmic transformation. Next, multivariable linear regression analysis (with logarithmic transformation) was performed to examine the associations between sociodemographic variables and total OOP medical expenditures among individuals with both hypertension and type 2 diabetes. We used the PROC SURVEYREG procedure and employed the exponential of the beta coefficient (*𝐸𝑥𝑝[β]*) to interpret the relationships between total OOP medical expenditures and sociodemographic variables. *𝐸𝑥𝑝(β)* that is less than 1 suggests relatively lower OOP medical expenditures for that subgroup compared to the reference group of the same factor while *𝐸𝑥𝑝(β)* that is higher than 1 suggests a relatively higher OOP medical expenditures in that subgroup compared to the reference group.

Finally, all analyses accounted for the MEPS complex survey design, including stratification (STRA9623), clustering (PSU9623), and person-level weighting (PERWT18F–PERWT22F). Because we pooled five years of MEPS data (2018–2022), we divided the person-level full-year weights by 5 to create an adjusted weight representing average annual estimates. Variances and 95% confidence intervals (CIs) were estimated using Taylor series linearization. All costs were inflation-adjusted to 2022 U.S. dollars. We used the U.S. Bureau of Labor Statistics Consumer Price Index (CPI) Inflation Calculator with December 2022 as the reference month. Each event-level spending component was adjusted to 2022 dollars before aggregating expenditures to the person level. All estimates were adjusted for all other sociodemographic variables in the regression analysis. All analyses were performed using SAS version 9.4, and *p*-value < 0.05 was considered statistically significant.

## Results

Table 1 presents the total OOP medical expenditures for adults with both hypertension and type 2 diabetes, categorized as combined, type 2 diabetes specific, and hypertension specific costs, between 2018 and 2022. For the combined costs, the unweighted minimum cost was $0.00, the weighted mean cost was $474.95, the unweighted median cost was $116.47, the unweighted maximum cost was $ 45,765.74, and the 95% CI of weighted mean: $424.62–$525.27. Similarly, the minimum, weighted average, median, maximum, and 95% CI of OOP medical expenditures for diabetes-specific costs were $0.00, $413.16, $65.88, $45,637.90, and 95% CI: $364.53–$461.80, respectively. The minimum, weighted average, median, maximum, and 95% CI of OOP medical expenditures for hypertension-specific costs were $0.00, $84.37, $19.68, $37,643.07, and 95% CI: $66.57–$102.19, respectively.

**Table 1:** Annual OOP medical expenditures among patients with both diabetes and hypertension: combined, diabetes-specific, and hypertension-specific costs

OOP cost type	Min	Mean	Median	Max	95%CI of Mean
Combined OOP cost	$0.00	$474.95	$116.47	$45,765.74	[424.62, 525.27]
Diabetes specific OOP cost	$0.00	$413.16	$65.88	$45,637.90	[364.53, 461.80]
Hypertension specific OOP cost	$0.00	$84.37	$19.68	$37,643.07	[66.57, 102.19]

Note: Each weighted spending component was adjusted to December 2022 U.S. dollars. Mean values were survey-weighted, whereas the minimum, median, and maximum values reflect unweighted sample observations. Diabetes-specific and hypertension-specific OOP costs were calculated separately. Medical events linked to both diabetes and hypertension were included in each condition-specific estimate but were counted only once in the combined OOP cost estimate. Therefore, the sum of the condition-specific costs may exceed the combined cost estimate

Table 2 depicts the unweighted descriptive characteristics of the analytic sample. Concerning age, 38% were between 50 and 64, while 62% were 65 years or older. Most individuals (44%) resided in the South, while 16% lived in the Northeast, 20% lived in the Midwest, and 20% lived in the West. The sample was predominantly female (53%) compared to males (47%). Regarding marital status, 47% of the sample were married, 43% were widowed, divorced, or separated, and 10% were never married. The racial composition included 53% non-Hispanic White, 5% Asian, 18% Hispanic, 21% Black or African American, and 3% in other or two more racial categories. The education levels include 24% with less than a high school degree, 33% with a high school degree, and 43% with some college experience or a college degree. Regarding employment status, 26% were employed, and 74% were unemployed. Family income levels were reported as follows: 29% of the sample were poor/near poor (< 125% FPL), 18% were low income (125%–199% FPL), 26% were middle income (200–399% FPL), and 27% were high income (≥ 400% FPL).

**Table 2:** Unweighted descriptive statistics and weighted multivariable linear regression results for OOP medical expenditures among individuals with both hypertension and type 2 diabetes

Statistical description		Weighted multivariable linear regression		
	*n* (%)	*Exp* [β] (SE)	95%CI	*p*-value
Age				
50–64 65+	2,755 (37.53) 4,586 (62.47)	Ref 1.26 (0.11)	– [1.01, 1.57]	– 0.04
Region				
Northeast Midwest South West	1,161 (15.82) 1,500 (20.43) 3,226 (43.94) 1,454 (19.81)	Ref 0.75 (0.10) 0.67 (0.10) 0.56 (0.10)	– [0.62, 0.91] [0.56, 0.81] [0.51, 0.76]	– < 0.01 < 0.001 < 0.001
Biological Sex				
Female Male	3,876 (52.80) 3,465 (47.20)	Ref 1.00 (0.10)	– [0.88, 1.14]	– 0.98
Race/ethnicity				
Asian Black or African American Non-Hispanic White Hispanic Others and two more	334 (4.55) 1,551 (21.13) 3,996 (53.77) 1,305 (21.13) 245 (3.34)	0.37 (0.21) 0.82 (0.09) Ref 0.57 (0.10) 0.54 (0.27)	[0.24, 0.56] [0.69, 0.98] – [0.46, 0.68] [0.32, 0.92]	< 0.001 0.03 – < 0.001 0.02
Marital Status				
Married Widowed/divorced/ separated Never married	3,454 (46.45) 3,139 (42.76) 748 (10.19)	Ref 0.82 (0.08) 0.76 (0.11)	– [0.70, 0.96] [0.61, 0.93]	– 0.01 < 0.01
Education				
Less than high school High school Some college/college degree	1,863 (25.06) 2,389 (32.54) 3,179 (43.30)	Ref 1.24 (0.10) 1.43 (0.09)	– [1.02, 1.52] [1.19, 1.71]	– 0.04 < 0.01
Employment Status				
Employed Unemployed	1,939 (26.41) 5,402 (73.59)	Ref 1.11 (0.10)	– [0.92, 1.34]	– 0.28
Insurance status				
Uninsured Medicare only Medicaid only/TRICARE only Private insurance only More than one insurance	348 (4.74) 2,588 (35.25) 538 (7.33) 1,228 (16.73) 2,639 (35.95)	2.08 (0.21) Ref 0.20 (0.18) 2.14 (0.15) 0.76 (0.09)	[1.37, 3.16] – [0.14, 0.28] [1.61, 2.85] [0.65, 0.90]	< 0.01 – < 0.001 < 0.001 < 0.01
Family Income Level				
Poor/near poor (< 125% FPL) Low income (125% − 199% FPL) Middle income (200–399% FPL) High income ((≥ 400% FPL)	2,205 (28.81) 1,289 (17.56) 1,894 (25.80) 2,043 (27.83)	Ref 1.51 (0.10) 1.76 (0.10) 2.17 (0.10)	– [1.25, 1.83] [1.46, 2.14] [1.78, 2.63]	– < 0.001 < 0.001 < 0.001
Perceived health status				
Excellent/ very good/good Fair Poor	4,743 (64.61) 1,905 (25.95) 693 (9.44)	Ref 1.16 (0.08) 1.33 (0.12)	– [0.99, 1.36] [1.05, 1.68]	– 0.07 0.02
Perceived mental health status				
Excellent/ very good/good Fair Poor	6,169 (84.03) 948 (12.91) 254 (3.46)	Ref 0.87 (0.13) 1.16 (0.19)	– [0.67, 1.13] [0.79, 1.71]	– 0.28 0.43
Total sample	7,341			

Note. FPL = Federal Poverty Line; SE= Standard error; CI = Confidence Interval. Ref = reference group. Values inside brackets represent percentages in the statistical description

For the insurance type, 5% had no insurance, 35% had Medicare only, 7% had Medicaid only or TRICARE only, 17% had private insurance, and 36% had more than one type of insurance. General and mental health status were reported by the respondent, who rated the general and mental health of each family member using the following categories. Among these participants, 65% reported excellent, very good, or good general health, 26% reported fair general health, and 9% reported poor general health. For mental health, 84% reported excellent, very good, or good mental health, 13% reported fair mental health, and 3% reported poor mental health.

Table 2 also presents the results of the adjusted model. We found no statistically significant differences in OOP expenditures by gender, employment status, or perceived mental health status. Compared with adults aged 50–64, those aged 65 + had OOP medical expenditures that were 1.26 times as high (*Exp[β]* = 1.26; 95% CI: 1.01–1.57; *p* = 0.04). Compared to individuals living in the Northeast region, residents in the Midwest (*𝐸𝑥𝑝[β]* = 0.75; 95%CI: 0.62–0.91; *p* < 0.01), the South (*𝐸𝑥𝑝[β]* = 0.67; 95%CI: 0.56–0.81; *p* < 0.001), and the West (*𝐸𝑥𝑝[β]* = 0.56; 95%CI: 0.51–0.76; *p* < 0.001) had lower OOP costs. Compared to individuals identifying as non-Hispanic White, Asians (*𝐸𝑥𝑝[β]* = 0.37; 95%CI: 0.24–0.56; *p* < 0.001), Black or African American groups (*𝐸𝑥𝑝[β]* = 0.82; 95%CI: 0.69–0.98; *p* = 0.03), Hispanics (𝐸𝑥𝑝*[β]* = 0.57; 95%CI: 0.46–0.68; *p* < 0.001), and individuals of other or two or more racial groups (*𝐸𝑥𝑝[β]* = 0.54; 95%CI: 0.32–0.92; *p* = 0.02) had lower OOP costs. Compared to those who were married, individuals who were widowed, divorced, or separated (*𝐸𝑥𝑝[β]* = 0.82; 95%CI: 0.70–0.96; *p* = 0.01) and those who had never been married (*𝐸𝑥𝑝[β]* = 0.76; 95%CI: 0.61–0.93; *p* < 0.01) had lower OOP costs. Compared with individuals with less than a high school education, those with a high school degree (*𝐸𝑥𝑝[β]* = 1.24; 95% CI: 1.02–1.52; *p* = 0.04) and those with some college or a college degree (*𝐸𝑥𝑝[β]* = 1.43; 95% CI: 1.19–1.71; *p* < 0.01) had higher OOP costs. Compared to those with Medicare, uninsured individuals (*𝐸𝑥𝑝[β]* = 2.08; 95% CI: 1.37–3.16; *p* < 0.01) had OOP costs, as did those with private insurance only (*𝐸𝑥𝑝[β]* = 2.14; 95% CI: 1.61–2.85; *p* < 0.001). However, those with Medicaid only or TRICARE only (*𝐸𝑥𝑝[β]* = 0.20; 95% CI: 0.14–0.28; *p* < 0.01) had lower OOP costs, as did those with more than one type of insurance (*𝐸𝑥𝑝[β]* = 0.76; 95% CI: 0.65–0.90; *p* < 0.01). Compared to households in the poor or near-poor income group, those with low income (*𝐸𝑥𝑝[β]* = 1.51; 95% CI: 1.25–1.83; *p* < 0.001), middle income (*𝐸𝑥𝑝[β]* = 1.76; 95% CI: 1.46–2.14; *p* < 0.001), and high income (*𝐸𝑥𝑝[β]* = 2.17; 95% CI: 1.78–2.63; *p* < 0.001) had higher OOP costs. Finally, patients with poor perceived health status (*𝐸𝑥𝑝[β]* = 1.33; 95% CI: 1.05–1.68; *p* = 0.02) have higher odds of incurring OOP costs.

**Fig. 1:**
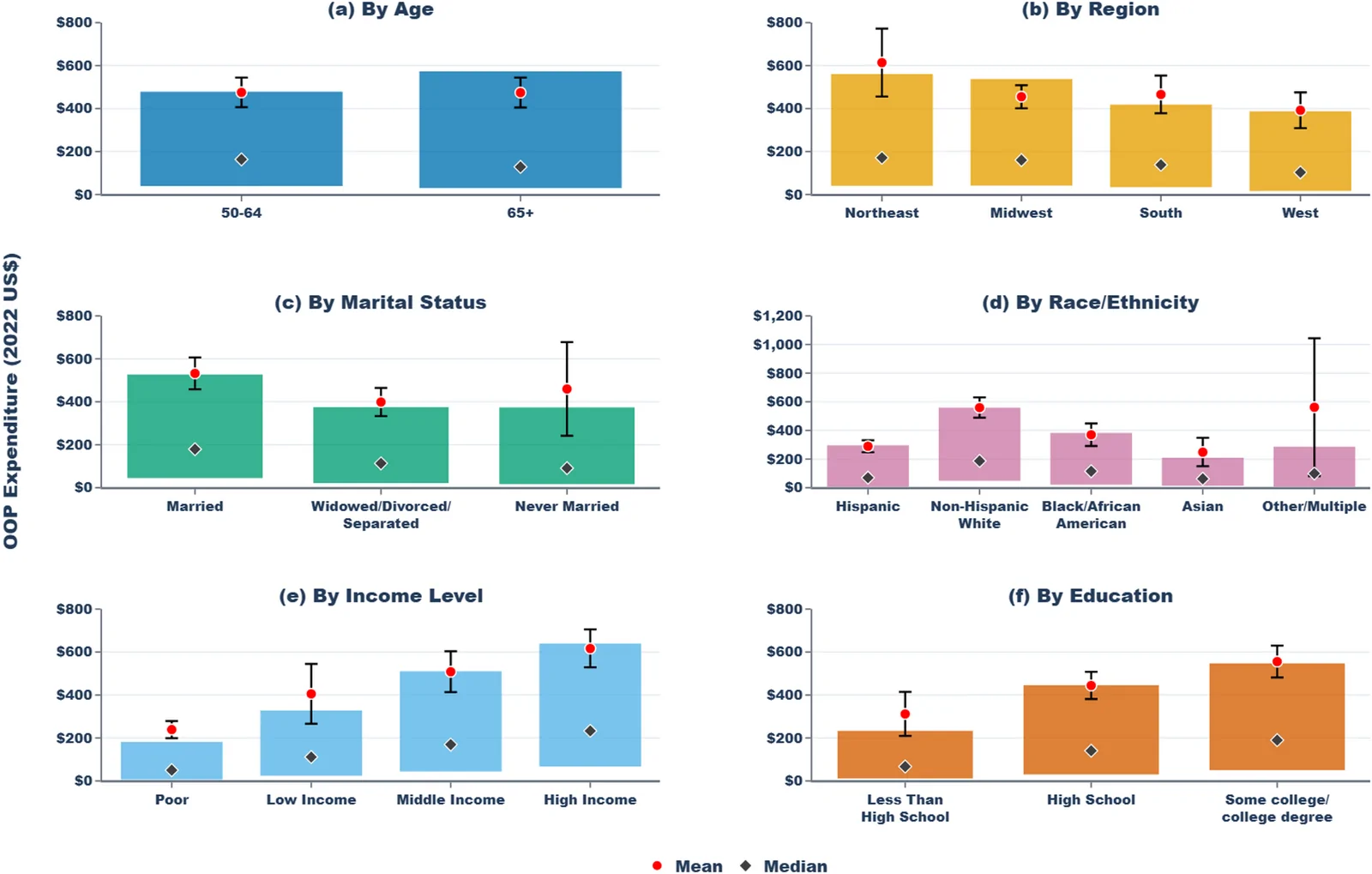
Results of the first quartile, median, mean, third quartile, and 95% CI of weighted OOP medical costs for significant demographic variables. Note. The red circle indicates the weighted mean OOP cost, while the blue diamond symbol indicates the weighted median OOP cost. Each weighted spending component was adjusted to December 2022 U.S. dollars

Figure 1. The first quartile is represented by the bottom of the light blue box, the median by the blue diamond symbol, the mean by the red circle symbol, and the third quartile by the top of the light blue box. The black vertical line indicates the 95% CI for weighted OOP medical costs by (a) age, (b) region, (c) race/ethnicity, (d) marital status, (e) annual family income and (f) education. This figure illustrates that weighted OOP costs exhibit a right-skewed distribution, with participants identifying as other/multiple races and those who were never married showing notably high outlier OOP cost values.

Weighted median OOP healthcare expenditures were $163.44 for adults aged 50–64 and $128.54 for adults aged 65 years and older. Regarding region, the weighted median OOP healthcare expenditures were $171.11 in the Northeast, $160.22 in the Midwest, $138.30 in the South, and $103.00 in the West. By marital status, the weighted median OOP costs were $179.51 for married individuals, $113.49 for widowed/divorced/separated individuals, and $91.17 for those who were never married. With respect to race/ethnicity, the weighted median OOP costs were $69.90 among Asian adults, $ 115.22 among Black/African American individuals, $186.77 among non-Hispanic White adults, $62.08 among Hispanic adults, and $99.34 among those identifying as other or multiple races. Across family income levels, the weighted median OOP costs increased with income, from $50.19 among poor/near-poor individuals to $111.35 among those with low income, $169.52 among those with middle income, and $232.76 among those with high income. Regarding education level, median OOP expenditures were $66.81 for individuals with less than a high school education, $140.42 for those with a high school degree, and $189.72 for those with some college or a college degree.

## Discussion

This study examined socioeconomic factors associated with variations in annual total OOP healthcare expenditures among a nationally representative sample of U.S. adults aged 50 years and older with both conditions. Key findings reveal several notable associations. Among patients with both conditions, type 2 diabetes accounted for substantially higher OOP costs compared with hypertension. Age, family income, region, educational attainment, race/ethnicity, marital status, perceived general health status, and insurance type were associated with higher OOP costs.

Poorer health status, as reported by the individual or another family member, was associated with higher OOP expenses. Additionally, a clear income gradient existed, with higher-income families spending more OOP cost on healthcare. Education attainment was associated with higher total OOP costs. Geographic and racial/ethnic disparities further highlighted regional cost variations and differential healthcare spending patterns across racial groups. Marital status and insurance type also played significant roles: unmarried individuals and those enrolled in Medicaid or TRICARE experienced lower OOP costs, while those with private insurance or no insurance faced higher OOP expenses. Collectively, these findings underscore that OOP spending is not determined by any single factor but is shaped by a complex network of associations in which socioeconomic characteristics, insurance coverage, and healthcare utilization collectively influence financial outlays.

Insurance type emerged as one of the strongest determinants of OOP expenditures, with Medicaid and TRICARE beneficiaries incurring lower OOP costs compared with those with private insurance. This likely reflects stronger cost-sharing protections within these public programs; however, lower OOP spending should not be interpreted as absence of affordability concerns, as many Medicaid beneficiaries face constrained incomes, limited provider availability, and other access barriers that may suppress utilization. Consistent with prior work, these findings align with prior work by Allen et al., which concluded that, on average, Medicaid enrollees had OOP expenses of $7.27, compared to $106.21 among their counterparts with commercial insurance, who are often exposed to deductibles and copayments for ongoing chronic disease management [16]. Additionally, we observed reduced OOP burden among individuals with multiple sources of coverage (e.g., Medicare and Medicaid), which may provide partial financial protection, although coverage coordination and service limits remain important considerations.

Perceived health status also played a significant role in OOP expenses, as individuals who reported poor health incurred higher OOP costs than those who reported excellent health. These findings align with those of two prior studies by Axon and Barrios (2022) [17] and Axon and Kamel (2021) [18]. In both evaluations of medical expenditures among older adults with pain, it was concluded that those who reported excellent health status had lower annual expenditures than those who reported fair or poor health. In addition, general health status reflects the severity and overall burden of diabetes and/or hypertension. Patients with these conditions are more likely to develop additional severe medical conditions, such as microvascular and macrovascular complications [19, 20] and disabilities [21], which can further increase OOP healthcare costs. Mistrust and limited access to healthcare may have been influential factors associated with differing perceptions of self-reported health [22, 23]. We posit a vicious cycle in which patients with hypertension and diabetes who report poor health incur additional healthcare expenses, leading to financial stress. This stress may have prompted them to adopt suboptimal disease management strategies in an attempt to maximize their limited resources, ultimately worsening their health outcomes and reinforcing their poor self-reported health.

Sociodemographic factors significantly influence OOP expenditures for individuals with chronic diseases. Individuals aged 65 and older incurred higher OOP costs. As age increases, older patients are at higher risk of developing multiple comorbidities. Individuals aged 65 years and older incur higher OOP cost when they have chronic conditions such as type 2 diabetes or hypertension [24]. Compared to the Northeast, individuals with both diabetes and hypertension living in the South, West, and Midwest were associated with lower OOP medical costs. In general, individuals in the Northeast tend to have higher family incomes, which may lead to greater healthcare utilization and, consequently, higher OOP costs. For example, the Northeast had the highest total diabetes-attributable annual economic costs per person with diabetes, as well as the highest total diabetes-attributable yearly medical costs per person with diabetes [25]. In addition, a 2024 study calculated the medical cost of diabetes for each U.S. state in 2022, with the Northeast having the highest estimated medical cost per population [6]. These results indicate that the Northeast may experience higher OOP costs relative to other regions.

Family income also plays an important role in individuals’ OOP costs, showing a clear dose response relationship in which higher income levels are associated with greater OOP expenditures. Although individuals with lower income may be eligible for Medicaid, which can reduce their OOP spending, another reason for their lower OOP costs may be reduced healthcare utilization. Low-income groups often face financial and structural barriers that limit access to healthcare services, resulting in lower overall OOP expenditures. For example, people with high incomes incurred the highest total OOP costs, while individuals with low incomes had lower overall OOP expenses but experienced a greater financial burden relative to their income [26]. The association between higher educational attainment and higher OOP costs likely operates indirectly, potentially through pathways such as securing employment with more comprehensive private insurance, which facilitates greater healthcare utilization, rather than implying a direct effect. Compared with individuals with less than a high school education, those with a high school education and those with some college or a college degree incurred higher OOP expenses. This finding aligns with previous research suggesting that individuals with higher education levels tend to have greater OOP costs [26, 27]. Additionally, Non-Hispanic White individuals with co-occurring diabetes and hypertension had the highest OOP medical costs compared to Asian, Hispanic, Black or African American, and other or two more racial groups, aligning with prior work [7, 28]. Higher OOP costs among individuals with higher socioeconomic status and non-Hispanic White adults are likely a reflection of better access to and utilization of healthcare services.

Never-married and widowed/divorced/separated individuals with both diabetes and hypertension had lower OOP medical costs compared to married individuals. One possible explanation is that family insurance plans often have higher OOP costs, including deductibles, than individual plans. Our findings align with prior work showing that individuals who have never married or are currently unmarried tend to have lower OOP costs [29]. This finding suggests that marital status may influence OOP healthcare costs through differences in insurance plan structures and cost-sharing mechanisms. These factors collectively enable married individuals to better access and utilize necessary healthcare services including preventive care and chronic disease management leading to higher overall utilization and thus higher OOP costs. Consequently, the lower OOP costs observed among unmarried individuals may not necessarily indicate lower healthcare needs or more favorable insurance coverage. Instead, they may partly reflect lower healthcare utilization or delayed use of necessary services because of more limited financial and social resources. Although married individuals bear higher absolute OOP costs, their typically higher household income mitigates the relative financial burden, underscoring the complex interplay between marital status, healthcare engagement, and economic capacity.

## Limitations

The present study has limitations in the reliability of the reported data. The data analyzed in the study were primarily self-reported and, therefore, subject to common method bias or possible confounding variables. However, the utilization of data from the MEPS has been well documented as the basis for understanding the average cost of healthcare for various populations. Therefore, the potential bias within the data should not sway the conclusions found within the present study. The cross-sectional nature of our data is a key limitation. It precludes any analysis of causal pathways or the shared etiology between diabetes and hypertension. Therefore, our findings should be interpreted as revealing associations between patient characteristics and financial burden, rather than establishing causality. Additionally, we use OOP cost as the outcome variable. While OOP expenses can reflect a family’s medical expenditures related to type 2 diabetes and hypertension, they do not fully capture the overall economic burden of medical costs experienced by the household. Our analytical sample included only adults aged 50 and older with both conditions. Therefore, our findings are not directly applicable to younger adult populations under 50. In future studies, we plan to extend the analysis to additional years of MEPS data and to examine pooled samples across the full age range.

## Conclusion

This study identified significant socioeconomic factors associated with OOP healthcare expenditures for the treatment of co-occurring type 2 diabetes and hypertension among U.S. individuals aged 50 years and older. Policymakers, healthcare providers, and healthcare administrators may find these findings useful as they implement solutions to manage chronic conditions such as diabetes and hypertension. Further studies are needed to build upon these findings and identify possible solutions to alleviate or reduce the influence of socioeconomic factors on combined OOP medical costs.

## Electronic Supplementary Material

Below is the link to the electronic supplementary material.

## Data Availability

The data used in this research were sourced from the Agency for Healthcare Research and Quality for the years 2018–2022. This publicly available data can be accessed through the Medical Expenditure Panel Survey (MEPS) website.

## Abbreviations

OOP: Out-of-pocket
MEPS: Medical Expenditure Panel Survey
CPI: Consumer Price Index
FPL: Federal poverty level
